# Effects of Tributyrin Supplementation on Growth Performance, Intestinal Digestive Enzyme Activity, Antioxidant Capacity, and Inflammation-Related Gene Expression of Large Yellow Croaker (*Larimichthys crocea*) Fed with a High Level of *Clostridium autoethanogenum* Protein

**DOI:** 10.1155/2023/2687734

**Published:** 2023-02-06

**Authors:** Xiuneng Wang, Min Wan, Zhen Wang, Haitao Zhang, Si Zhu, Xiufei Cao, Ning Xu, Jichang Zheng, Xianyong Bu, Wei Xu, Kangsen Mai, Qinghui Ai

**Affiliations:** ^1^Key Laboratory of Aquaculture Nutrition and Feed (Ministry of Agriculture and Rural Affair) and Key Laboratory of Mariculture (Ministry of Education), Ocean University of China, Qingdao 266003, China; ^2^Guangdong Evergreen Feed Industry Co., Ltd., Key Laboratory of Aquatic, Livestock and Poultry Feed Science and Technology in South China, Ministry of Agriculture and Rural Affairs, Zhanjiang 524000, China; ^3^Laboratory for Marine Fisheries Science and Food Production Processes, Qingdao National Laboratory for Marine Science and Technology, 1 Wenhai Road, Qingdao 266003, China

## Abstract

An 8-week growth experiment was conducted to investigate effects of tributyrin (TB) supplementation on growth performance, intestinal digestive enzyme activity, antioxidant capacity, and inflammation-related gene expression of juvenile large yellow croaker (*Larimichthys crocea*) (initial weight of 12.90 ± 0.02 g) fed diets with high level of *Clostridium autoethanogenum* protein (CAP). In the negative control diet, 40% fish meal was used as the major source of protein (named as FM), while 45% fish meal protein of FM was substituted with CAP (named as FC) to form a positive control diet. Based on the FC diet, grade levels of 0.05%, 0.1%, 0.2%, 0.4%, and 0.8% tributyrin were added to formulate other five experimental diets. Results showed that fish fed diets with high levels of CAP significantly decreased the weight gain rate (WGR) and specific growth rate (SGR) compared with fish fed the FM diet (*P* < 0.05). WGR and SGR were significantly higher than in fish fed diets with 0.05% and 0.1% tributyrin that fed the FC diet (*P* < 0.05). Supplementation of 0.1% tributyrin significantly elevated fish intestinal lipase and protease activities compared to FM and FC diets (*P* < 0.05). Meanwhile, compared to fish fed the FC diet, fish fed diets with 0.05% and 0.1% tributyrin showed remarkably higher intestinal total antioxidant capacity (T-AOC). Malondialdehyde (MDA) content in the intestine of fish fed diets with 0.05%-0.4% tributyrin was remarkably lower than those in the fish fed the FC diet (*P* < 0.05). The mRNA expressions of tumor necrosis factor *α* (*tnfα*), interleukin-1*β* (*il-1β*), interleukin-6 (*il-6*), and interferon *γ* (*ifnγ*) were significantly downregulated in fish fed diets with 0.05%-0.2% tributyrin, and the mRNA expression of *il-10* was significantly upregulated in fish fed the 0.2% tributyrin diet (*P* < 0.05). In regard to antioxidant genes, as the supplementation of tributyrin increased from 0.05% to 0.8%, the mRNA expression of nuclear factor erythroid 2-related factor 2 (*nrf2*) demonstrated a trend of first rising and then decreasing. However, the mRNA expression of Kelch-like ECH-associated protein 1 (*keap1*) was remarkably lower in fish fed the FC diet than that fed diets with tributyrin supplementation (*P* < 0.05). Overall, fish fed tributyrin supplementation diets can ameliorate the negative effects induced by high proportion of CAP in diets, with an appropriate supplementation of 0.1%.

## 1. Introduction

As the major source of protein, fish meal is widely used in fish feed formulation for its nice palatability and balanced nutrition. However, the conflict between high demand for fish meal and shortage of fish meal supply has seriously restricted aquaculture development [1, 2]. The aquaculture feed industry has spared no effort to find alternative sources of proteins that are suitable, low-cost, and available for substituting fishmeal. *Clostridium autoethanogenum* protein (CAP) is a novel bacterial protein produced by *Clostridium autoethanogenum* (without toxic genes) [3] fermented with carbon monoxide (CO). As a type of single-cell protein (SCP), CAP has pleasant quality of high protein content and balanced profile of amino acids, showing great potential to replace fish meal. The studies on largemouth bass (*Micropterus salmoides*) (CAP replacement level ≤ 43%) and juvenile turbot (*Scophthalmus maximus L*.) (CAP replacement level ≤ 45%) suggested that an appropriate level of CAP replacing fish meal did not have a significant negative effect on growth performance [4, 5]. However, fish growth performance was compromised by a high level of CAP replacing fish meal in diets. For example, Jiang et al. [6] found that the Pacific white shrimp (*Litopenaeus vannamei*) growth was significantly reduced and intestinal morphology and immunity were negatively affected as the CAP replacement ratio was higher than 45%. Similarly, excessive CAP substitution (≥45%) had a significant adverse impact on the growth of large yellow croaker (*Larimichthys crocea*) [7].

Due to the implementation of relevant policies to reduce or eliminate use of antibiotics, research on harmless additives has gradually received more attention [8]. Butyric acid, a type of short-chain fatty acid, is considered to be a promising feed additive owing to its positive role in production applications [9]. Butyrate was initially identified as the main energy source for the intestines [10]. Moreover, butyrate can regulate inflammation and intestinal barrier function through inhibition of histone deacetylases and interactions with G protein-coupled receptors [11]. The multiple beneficial effects on the intestine of butyrate are well documented, which proved that butyrate plays a regulatory role in immune regulation, enhances epithelial defense barrier, and ameliorates oxidative stress and mucosal inflammation, transepithelial fluid transport, intestinal motility, and visceral perception [12]. However, the unpleasant odor of butyrate limits the consumption of butyrate-containing feeds [13], and butyrate is often absorbed prematurely in the digestive tract rather than reach the colon to function [14]. For better application of butyrate in fish species, some production forms including butyric acid (BA), butyrate glycerides (BG), microencapsulated sodium butyrate (MSB), sodium butyrate (SB), and tributyrin (TB) were developed [15]. Tributyrin is composed of a glycerol backbone and three butyric acid lipid molecules, which have higher stability and produce more butyric acid in the gut compared to other butyrate forms [16]. Tributyrin has been proved to regulate gut health and inflammation. Previous studies found that tributyrin supplementation attenuated ethanol-induced intestinal inflammation in mice [17] and similar studies in piglets [18]. In regard to aquatic animals, studies also reported positive effects of tributyrin on growth performance, intestinal morphology, microbiota, and lipid metabolism of tributyrin in black sea bream (*Acanthopagrus schlegelii*) [19], snake head (*Channa argus*) [20], large yellow croaker [21], and yellow drum (*Nibea albiflora*) [22].

Large yellow croaker is the major economic fish widely farmed in southeast coastal areas of China [23]. To date, extensive studies have been conducted on large yellow croaker in the nutritional requirements and replacement of fish meal [24–26]. Based on the research on carnivorous fish including largemouth bass [4, 27], turbot [5], and large yellow croaker [7], we supposed that 45% may be a high level of CAP replacement. To make CAP more widely applied, growth performance, intestinal digestive enzyme activity, antioxidant capacity, and inflammation-related gene expression were evaluated in large yellow croaker fed with different levels of tributyrin supplementation in a high-CAP diet.

## 2. Materials and Methods

### 2.1. Ethical Approval

The use and care of animals were approved by the Committee on the Ethics of Animal Experiments of Ocean University of China and followed by Management Rule of Laboratory Animals (Chinese Order NO.676 of the State Council, revised 1 March 2017).

### 2.2. Experimental Diets

Seven isonitrogenous and isolipid diets (containing 42% crude protein and 12% crude lipid) were formulated in this experiment. Fish oil and soybean lecithin were the main source of lipid, and bread flour was the main source of carbohydrate in diets. As the main protein source, the composition of CAP and fish meal was analyzed (Table 1). The negative control diet named as FM was designed with inclusion of 40% fish meal, while CAP was used replacing 45% fish meal protein in the positive control named as FC. The amino acid compositions of the FM and FC diets were analyzed (Table 2). Based on FC, other 5 experimental diets supplementing graded levels of 0.05%, 0.1%, 0.2%, 0.4%, and 0.8% tributyrin (Shanghai Menon Animal Nutrition Technology Co., Ltd., Shanghai, China) were formulated (Table 3). In brief, the raw ingredients were thoroughly ground and sieved. The tributyrin was blended into bread flour as premix. All ingredients were mixed with liquid mixture of fish oil, soybean lecithin, and clean water to produce a stiff dough and then using a granulator to produce the pellets (3 mm × 5 mm). All pellets were dried at 50°C overnight then refrigerated at -20°C before use.

### 2.3. Feeding Procedure

All juvenile large yellow croakers were supplied by Aquatic Seeds Farm of the Marine and Fishery Science and Technology Innovation Base, Ningbo, China. Before the experiment, juveniles were fed commercial diets and acclimated in a floating sea cage (4 m × 8 m × 4 m) for 14 days. After acclimation, juveniles (initial body weight: 12.90 ± 0.02 g) were randomly apportioned to 21 sea cages (1 m × 1 m × 2 m). Each experimental diet was allocated to three cages containing 40 fish per cage. Juveniles were hand-fed two times a day (05:00 and 17:00) until visual apparent satiation for 8 weeks and growth with appropriate water environmental conditions (temperature: 18.4 to 24.2°C; dissolved oxygen level: 6.3 to 7.6 mg/L, and salinity: 26.5 to 29.3‰) during the experimental period.

### 2.4. Sampling

At the end of the experiment, juveniles per cage were anesthetized with eugenol (1: 10,000; Shanghai Reagent, China) after being starved for one day. The number of total fish in each cage was counted, and final body weight was recorded to detect survival rate and growth performance. The wet weight of the body, viscera, and liver was measured to calculate the morphological indexes. Four fish of each cage were randomly selected and refrigerated at -20°C for whole-body composition analysis. Six fish of each cage were sampled for serum samples and intestine samples for further analysis. Use a 1 mL syringe to collect blood from the fishtail vein, refrigerate at 4°C overnight, and then centrifuge (4,000 rpm for 15 min) to collect serum. Serum and intestine samples were put into liquid nitrogen immediately then stored at –80°C for further analysis.

### 2.5. Experimental Diets and Whole Fish Body Composition Analysis

Moisture, crude protein, and crude lipid in the whole fish body were analyzed by AOAC (1995). Moisture was measured by drying samples at 110°C to a constant weight. Crude protein was measured based on the Kjeldahl method, and crude lipid was measured by the Soxhlet extraction method (FOSS, Soxtec 2050).

The freeze dryer (ALPHA 1–4 LD freeze dryer, Kleist, Germany) was used to dry the raw materials and experimental diets to a constant weight at –50°C. The automatic amino acid analyzer (L-8900, Hitachi) was used to determine the amino acid profiles of freeze-dried samples after acid hydrolysis (6 N HCl at 110°C for 24 h).

### 2.6. Serum Index Analysis

The serum alkaline phosphatase (ALP), alanine transaminase (ALT), and aspartate transaminase (AST) were detected by commercial kits provided by Nanjing Jiancheng Bioengineering Institute (Nanjing, China). The preparation and operation of the reagents were carried out strictly in accordance with the operating procedures of the specific kits.

### 2.7. Intestinal Digestive Enzymes and Antioxidant Activity

The intestinal digestive enzyme activity of amylase, trypsin, and lipase and the intestine antioxidant capacity index of total antioxidant capacity (T-AOC), superoxide dismutase (SOD), malondialdehyde (MDA), and catalase (CAT) were assessed by specific commercial kits. All kits were obtained from Nanjing Jiancheng Bioengineering Institute (Nanjing, Jiangsu, China). The preparation and operation of the reagents were carried out according to the operating procedures of the specific kits.

### 2.8. RNA Extraction and Real-Time Quantitative PCR

Total RNAs of intestine were extracted in strict accordance with the instruction of RNA isolation kit (Vazyme, Nanjing, China). A NanoDrop 2000 spectrophotometer (Thermo Fisher Scientific, USA) was used to detect the quality and concentration of RNA. HiScript II Q RT SuperMix for qPCR (+gDNA wiper) purchased from Vazyme (Nanjing, China) was used to reversely transcribe RNA to cDNA in strict accordance with the instruction of manufacturer. The CFX Connect Real-Time System (Bio-Rad) was used to analyze relative quantifies of target genes; then, a total volume of qPCR was carried out as follows: 1 *μ*L cDNA primer, 2 *μ*L cDNA product, 10 *μ*L ChamQ SYBR qPCR Master Mix (Vazyme, Nanjing, China), and 7 *μ*L RNAase-free water. Primers used in this study are shown in Table 4. The RT-qPCR program was set according to previous research [28]. The amplification efficiency and specificity of the product were confirmed by a standard curve and melting curve analysis. The gene expression levels were analyzed using the 2^−*ΔΔ*CT^ methods.

### 2.9. Calculations and Statistical Methods

(1) $$\begin{array}{c} \text{Weight gain rate}\left(\text{WGR},\%\right)=100\times \frac{W_{t}-W_{o}}{W_{o}},\\ \text{Specific growth rate}\left(\text{SGR},\%/\text{d}\right)=100\times \frac{\text{Ln}W_{t}-\text{Ln}W_{o}}{d},\\ \text{Hepatosomatic index}\left(\text{HSI},\%\right)=100\times \frac{\text{liver weight}}{\text{body weight}},\\ \text{Viscerasomatic index}\left(\text{VSI},\%\right)=100\times \frac{\text{visceral weight}}{\text{body weight}},\\ \text{Feed intake}\left(\text{FI},\%/\text{day}\right)=100\times \frac{F}{d\times \left({\text{W}}_{\text{t}}+{\text{W}}_{\text{o}}\right)/2},\\ \text{Feed efficiency ratio}\left(\text{FER}\right)=\frac{\text{wet weight gain}}{F},\\ \text{Protein efficiency rate}\left(\text{PER},\%\right)=\frac{\text{wet weight gain}}{\text{total protein fed}},\\ \text{Survival rate}\left(\text{SR},\%\right)=100\times \frac{\text{FN}}{\text{IN}}, \end{array}$$

where *W*_*t*_ was the final weight and *W*_*o*_ was the initial weight of fish in the cage, *d* was the experiment period, *F* was the total feed consumption (g, dry matter), and FN and IN were the final and initial numbers of fish.

SPSS Statistics 25.0 software (IBM, USA) was used to analyze the data by using one-way analysis of variance (ANOVA), and then, specific differences were assessed by Tukey's test. All the data were expressed as means ± SEM (standard error of the mean). The level of significant difference was indicated by *P* < 0.05.

## 3. Result

### 3.1. Survival, Growth Performance, and Morphological Indexes

**Figure 1:**
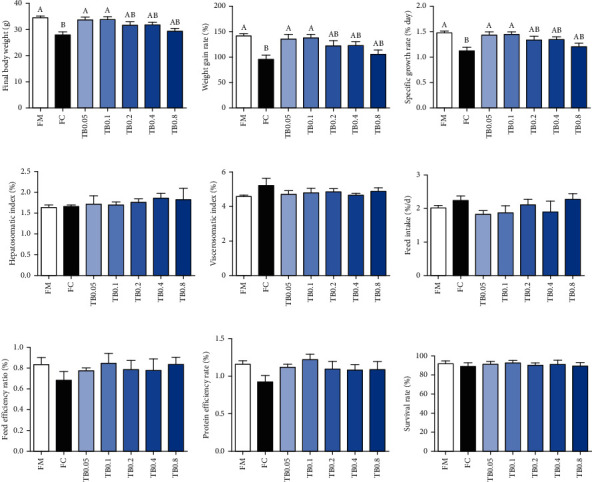
Effects of tributyrin supplementation on survival and growth performance of juvenile large yellow croaker fed diets with high level of *Clostridium autoethanogenum* protein replacing fish meal. Values are means (*n* = 3), with the vertical bars represent the standard error. Data in bars sharing the common letters indicated no significant difference determined by Tukey's test (*P* > 0.05).

SR ranging from 0.05% to 0.8% tributyrin showed no significant difference (*P* > 0.05). WGR and SGR in fish fed the FC diet were significantly lower compared with fish fed the FM diet. The supplementation of 0.05% and 0.1% tributyrin significantly increased the WGR and SGR compared to fish fed with the FC diet (*P* < 0.05). No remarkable differences were observed in HSI, VSI, and FER in all treatments (*P* > 0.05) (Figure 1).

### 3.2. Body Composition

No remarkable differences were detected in the moisture and crude protein of whole fish body in all tributyrin treatments (*P* >0.05). The highest whole body crude lipid content was observed in the fish fed with the FM diet, but not significantly different in all tributyrin treatments (*P* > 0.05) (Table 5).

### 3.3. Serum Biochemical Indexes

No remarkable differences were was observed in serum alkaline phosphatase (ALP) activity (*P* > 0.05). As the tributyrin level elevated from 0.05% to 0.8%, serum alanine transaminase (ALT) activity showed a first decreasing then increasing trend. The activity of serum ALT in fish fed diets with 0.05-0.2% tributyrin was significantly lower than that that in fish fed the FC diet. The activity of serum AST in fish fed tributyrin supplementation diets had significantly lower than that that in fish fed the FC diet (*P* < 0.05) (Table 6).

### 3.4. Intestinal Digestive Enzyme Activity

No remarkable differences were detected in the activity of amylase in all tributyrin treatments (*P* > 0.05). As the tributyrin level elevated from 0.05% to 0.8%, the activity of lipase showed increasing followed by decreasing trends. The activity of lipase was remarkedly higher in fish fed with 0.1% tributyrin than in fish fed the FC diet (*P* < 0.05). The activity of trypsin was remarkedly higher in fish fed diets with 0.05% and 0.1% tributyrin compared to those fed FM and FC diets (*P* < 0.05) (Table 7).

### 3.5. Intestinal Antioxidant Capacity and the mRNA Expression of Antioxidant-Related Genes in the Intestine

No remarkable differences were detected in T-AOC, SOD, and CAT between fish fed with FM and FC diet (*P* > 0.05). The activity of T-AOC was significantly increased in fish fed with 0.05% tributyrin than that in the FC diet, while the activity of SOD was significantly increased in fish fed with 0.1% tributyrin than that in the FC diet (*P* < 0.05). The supplementation of 0.05%-0.4% tributyrin significantly decreased MDA content compared with fish fed with FC diet (*P* < 0.05). Moreover, the activity of CAT in fish fed the diet with 0.8% tributyrin was notably decreased compared to those fed with FC diet (*P* < 0.05) (Table 8).

**Figure 2:**
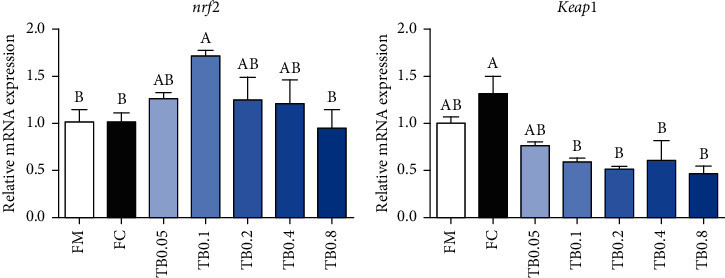
Effects of tributyrin supplementation on the expression of antioxidant-related genes in the intestine of juvenile large yellow croaker fed diets with high level of *Clostridium autoethanogenum* protein replacing fish meal. Values are means (*n* = 3), with the vertical bars represent the standard error. Data in bars sharing the common letters indicated no significant difference determined by Tukey's test (*P* > 0.05). *nrf2*: nuclear factor erythroid 2-related factor 2; *keap1*: Kelch-like ECH-associated protein 1.

In regard to antioxidant-related genes, with the tributyrin level elevated from 0.05% to 0.8%, mRNA expressions of *nrf2* showed increasing followed by decreasing trends. 0.1% tributyrin supplementation had notably increased the expression level of *nrf2* (*P* < 0.05). Besides, the expression levels of *keap1* were notably downregulated in fish fed with 0.1%-0.8% tributyrin diets compared with the FC diet (*P* < 0.05) (Figure 2).

### 3.6. The mRNA Expression of Inflammation-Related Genes in the Intestine

**Figure 3:**
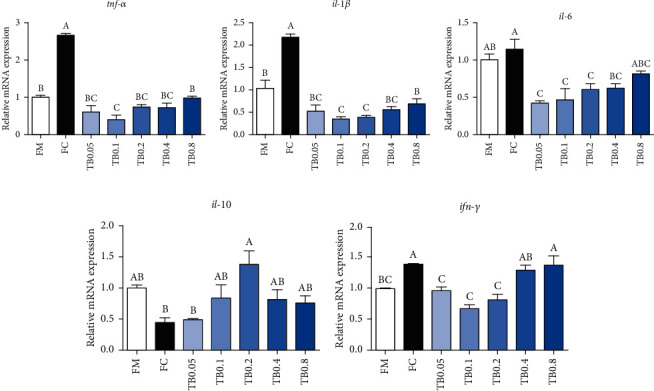
Effects of tributyrin supplementation on the expression of inflammation-related genes in the intestine of juvenile large yellow croaker fed diets with high level of *Clostridium autoethanogenum* protein replacing fish meal. Values are means (*n* = 3), with the vertical bars represent the standard error. Data in bars sharing the common letters indicated no significant difference determined by Tukey's test (*P* > 0.05). *tnfα*: tumor necrosis factor *α*; *il-1β*: interleukin-1*β*; *il-6*: interleukin-6; *il-10*: interleukin-10; *ifnγ*: interferon *γ*.

The expression of proinflammatory genes in fish fed the FC diet were upregulated compared to the FM diet. With the tributyrin level elevated from 0.05% to 0.8%, mRNA expressions of *tnf-α*, *il-1β*, and *ifn-γ* showed decreasing trends followed by increasing trends (*P* < 0.05). Compared to the FC diet, mRNA expression of *tnf-α* and *il-1β* was remarkably downregulated in fish fed with tributyrin (*P* < 0.05). The mRNA expressions of *ifn-γ* and *il-6* in fish fed the diet with 0.05-0.2% tributyrin were markedly lower than those in fish fed the FC diet (*P* < 0.05). Moreover, when the tributyrin level elevated from 0.05% to 0.8%, mRNA expression of anti-inflammatory genes *il-10* increased followed by decreased and was remarkably higher in fish fed with 0.2% tributyrin diet than those fed the FC diet (*P* < 0.05) (Figure 3).

## 4. Discussion

Tributyrin had a positive effect on the growth performance of large yellow croaker. In this study, substituting 45% of fish meal protein with CAP significantly degraded the growth of large yellow croaker, which indicated that high level of CAP replacing fish meal can have a negative effect on fish growth. This result ties well with previous studies in pacific white shrimp [6] and largemouth bass [27]. The decline in growth performance may be attributed to the lack of arginine in CAP and some unknown growth factors in fish meal [6]. However, the WGR and SGR were increased with the appropriate tributyrin supplementation (0.05% and 0.10%) in diet with high levels of CAP, indicating that the growth performance of large yellow croaker could be improved by tributyrin supplementation, which was consistent with previous research in our lab [21]. Similar results were found in marine fish including golden pompano [29], turbot [30], and sea bream [31]. A study in tawny puffer showed that WG, SGR, feed efficiency (FE), and protein efficiency ratio (PER) of fish were increased with the increasing TB level in diets [32]. In our study, tributyrin supplementation improved feed efficiency and protein utilization compared to FC, but did not reach a significant level. The difference could be due to different species, supplementation levels, and CAP replacement levels. Previous studies have shown that the supplementation of butyrate and its derivatives in the diets of animal production species including pigs [33], poultry [34], and ruminants [35, 36] facilitates the development of the gastrointestinal tract, promotes the digestion and absorption of nutrients, and improves the gut health of animals. The result of these improvements is often related to an observed increase in growth performance [13]. Our experiment also found that an appropriate amount of tributyrin can improve the digestive enzyme activity and antioxidant capacity of the intestine and reduce inflammation. However, dose-response experiments reported that high butyrate in diet had no positive effect or even had an adverse effect on the growth performance of fish [15]. A previous study on black sea bream showed that adverse effects on growth performance could be observed when the tributyrin supplementation reached 0.8% in the diet [19]. In our study, a similar result was observed. Presumably, high levels of butyrate products have a strong odor and bitter taste [37], which may reduce fish feed intake and reduce fish growth performance.

Digestive enzyme activity affects the feed utilization of fish, which is an important factor in optimizing dietary formula [38]. Previous studies have demonstrated the substitution of SCP for fish meal seems to have little effect on the digestive enzyme activity of aquatic animals. In pacific white shrimp, the activities of digestive enzymes did not significantly affect when diet fish meal replaced by SCP [39]. In the present study, the digestive enzyme activities in fish fed with 45% CAP were decreased without a significant difference compared with the FM diet. CAP has been proven to have good absorbing properties in fish. The apparent digestibility of CAP by largemouth bass was 82.77%, 87.44%, and 97.48%, respectively [40]. 45% CAP substitution ratio may not be enough to inhibit digestion and absorption of large yellow croaker. However, tributyrin supplementation significantly increased digestive enzyme activities compared with the FC diet. In intrauterine retarded piglets, tributyrin supplementation significantly increased lipase activity in the ileum and trypsin activity in the ileum and jejunum [33]. It was also reported that dietary supplementation of 2.5 g/kg tributyrin increased the activities of lipase and protease in snakehead [20]. As the major energy source for intestinal epithelial, butyric acid plays a vital role in absorption, feed digestion, and promoting intestinal development [41]. Supplementation of tributyrin may participate in releasing butyrate, thus promoting the absorption of CAP nutrients by fish to improve growth performance to some extent.

Further investigation showed the effect of tributyrin on enhancing antioxidant capacity. Oxidative stress results from an imbalance between the generation of oxygen-derived radicals and the antioxidant defenses of organism. Organisms have evolved a system to prevent and repair the impact of oxidative stress. Prevention comes in the antioxidant form, which can be enzymatic [42]. Antioxidant capacity is a crucial index for evaluating the health and oxidative stress status of fish. Tributyrin supplementation reduced MDA content and increased the activities of T-AOC and SOD in intestine. Results in this study showed that 45% CAP replacement of fish meal could cause slight oxidative damage to juvenile large yellow croaker intestine, and this negative effect could be alleviated by tributyrin supplementation. Moreover, at the gene level, our results indicated that the mRNA expression of the antioxidant-related gene *nrf2* was remarkably upregulated in the 0.1% tributyrin treatments, while the mRNA expression of *keap1* was downregulated. A previous study in goats [43] lead the consisted result suggesting that butyrate may increase the antioxidant enzyme activity by regulating the Nrf2 signaling pathway, thus alleviating oxidative damage. Sodium butyrate supplementation can also enhance the intestinal physical barrier function of grass carp through the Nrf2 signal pathway [44].

Oxidative stress is usually accompanied by elevated levels of inflammation; appropriate amounts of inflammatory factors are involved in damaged tissue regeneration, which can repair and heal wounds. Severe inflammation will impair tissues and cells, causing various inflammatory diseases and seriously compromise the normal immune performance of fish [45, 46]. Therefore, intestinal inflammatory status was assessed in this study. The result indicated that high level of CAP replacing fish meal could upregulate the expression of proinflammatory genes and downregulate the expression of anti-inflammatory genes, suggesting that the immune function of the intestine may be impaired. Similar findings were observed in the study of Pacific shrimp, in which 30% CAP replacement significantly upregulated the immune genes such as *cox1* and *cox2*, and the damage appeared to be more severe when the replacement level reached 70% [6]. Our results also found that tributyrin supplementation could remarkably suppress inflammation. The mRNA expression of genes related to anti-inflammatory cytokines *il-10* was significantly elevated in fish fed the tributyrin treatment diets compared to the FC diet, while expression of pro-inflammatory cytokines (*tnf-α*, *il-1β*, *il-6*, and *ifn-γ*) showed a reverse trend. In accordance with findings in our study, mammals reported that tributyrin reduces content of TNF-*α*, IL-6, and IL-1*β* in macrophages of mice fed a high-fat diet [47]. Wang et al. [18] found that intestinal inflammation and oxidative stress could be attenuated by tributyrin supplementation in diquat-challenged pigs. The anti-inflammatory properties of butyrate have also been reported in aquatic animals. Ding et al. [9] found tributyrin could significantly reduce lipid peroxidation and intestinal protein carbonylation levels and degrade *tnf-α* and *il-16* expression levels in the intestine of shrimp. A similar conclusion was found in grass carp [48] and common carp [49]. Moreover, the mechanism of this anti-inflammatory effect could be due to butyrate which plays an immunomodulatory role by inhibiting the activation of nuclear factor kappa B (NF-*κ*B) signaling pathway and then suppressed the initiation and expression of downstream proinflammatory cytokines and a group of chemokine genes [48, 50, 51]. Previous studies indicated NF-*κ*B activation may suppress the myosin light chain kinase (MLCK) expression, then enhance intestinal integrity, and reduce the permeability of the intestinal mucosal epithelium to pathogens to exert anti-inflammatory effects [22, 52].

## 5. Conclusion

In conclusion, this study indicated appropriate supplementation of tributyrin has a positive effect on the growth performance of large yellow croaker fed with a high level of CAP-replaced fish meal, which is mainly attributed to the enhancement of intestinal digestive enzyme activity and antioxidant capacity and reduced the inflammatory response of large yellow croaker. In terms of this study, the optimal tributyrin level was approximately 0.1% with CAP replaced 45% fish meal protein in a diet for juvenile large yellow croaker.

## Figures and Tables

**Table 1:** Compositions of fishmeal and the *Clostridium autoethanogenum* protein (CAP) used in the present study (%, dry matter).

	Fish meal	CAP
Arginine	4.35	3.23
Histidine	2.15	1.28
Isoleucine	2.70	5.50
Leucine	4.96	6.33
Lysine	5.15	8.49
Methionine	1.92	2.24
Phenylalanine	3.34	3.40
Threonine	2.60	4.10
Valine	3.12	5.32
Alanine	4.34	4.16
Aspartic acid	5.88	9.12
Cysteine	0.51	0.56
Glutamic acid	8.49	8.80
Glycine	3.93	3.98
Proline	2.59	2.32
Serine	2.49	3.05
Tyrosine	1.87	2.66

**Table 2:** Amino acid (AA) compositions of the FM and FC experimental diets (%, dry matter).

	FM	FC
Arginine	2.64	2.41
Histidine	1.35	1.11
Isoleucine	1.50	1.83
Leucine	3.28	3.50
Lysine	3.06	3.39
Methionine	0.33	0.48
Phenylalanine	2.08	2.08
Threonine	1.94	1.95
Valine	1.73	1.84
Alanine	2.70	2.56
Aspartic acid	4.05	4.30
Cysteine	0.34	0.34
Glutamic acid	7.08	7.07
Glycine	2.15	2.07
Proline	2.21	2.15
Serine	2.09	2.07
Tyrosine	1.46	1.50

**Table 3:** Formulation and proximate composition of the experiment diets (% dry weight).

Ingredients	Diets						
	FM	FC	TB0.05	TB0.1	TB0.2	TB0.4	TB0.8
Fish meal^1^	40.00	22.00	22.00	22.00	22.00	22.00	22.00
Krill meal^1^	3.00	3.00	3.00	3.00	3.00	3.00	3.00
Soybean protein concentrate^1^	12.00	12.00	12.00	12.00	12.00	12.00	12.00
Bread flour^1^	31.25	31.25	31.25	31.25	31.25	31.25	31.25
*Clostridium autoethanogenum* protein^1,2^	0	16.00	16.00	16.00	16.00	16.00	16.00
Pregelatinized starch	2.00	2.00	1.95	1.90	1.80	1.60	1.20
Fish oil	6.00	8.00	8.00	8.00	8.00	8.00	8.00
Choline chloride	0.20	0.20	0.20	0.20	0.20	0.20	0.20
Soybean lecithin	2.00	2.00	2.00	2.00	2.00	2.00	2.00
Vc polyphosphate	0.05	0.05	0.05	0.05	0.05	0.05	0.05
Ca(H_2_PO_4_)_2_	2.00	2.00	2.00	2.00	2.00	2.00	2.00
Vitamin premix^3^	0.20	0.20	0.20	0.20	0.20	0.20	0.20
Mineral premix^4^	1.00	1.00	1.00	1.00	1.00	1.00	1.00
Attractant^5^	0.20	0.2	0.2	0.2	0.2	0.2	0.2
Mould inhibitor	0.05	0.05	0.05	0.05	0.05	0.05	0.05
Ethoxy quinoline	0.05	0.05	0.05	0.05	0.05	0.05	0.05
Tributyrin^6^	0	0	0.05	0.1	0.2	0.4	0.8
Total	100.00	100.00	100.00	100.00	100.00	100.00	100.00
Analyzed nutrient composition (% dry matter basis)							
Crude protein	43.08	43.67	42.95	43.46	43.51	43.62	43.29
Crude lipid	11.38	11.45	11.27	11.86	11.54	11.78	11.72

^1^Fish meal (71.22% crude protein, 11.12% crude lipid); krill meal (53.18% crude protein, 13.10% crude lipid); soybean protein concentrate (64.77% crude protein); bread flour (17.39% crude protein, 0.34% crude lipid); *Clostridium autoethanogenum* protein (82.30% crude protein). ^2^*Clostridium autoethanogenum* protein was provided by Hebei Shoulang New Energy Technology Co., Ltd., Hebei, China. ^3^Vitamin premix (mg/kg diet), retinal acetate, 32; alpha-tocopherol, 240; menadione, 10; thiamin, 25; pyridoxine HCl, 20; vitamin B12, 10; riboflavin, 45; pantothenic acid, 60; cholecalciferol, 5; folic acid, 20; niacin 200; biotin, 60; inositol, 800; microcrystalline cellulose, 13473. Vitamin C was supplied in the form of vitamin C polyphosphate. ^4^Mineral premix (mg/kg diet), MgSO_4_·7H_2_O, 1200; FeSO_4_·H_2_O, 80; ZnSO_4_·H_2_O, 50; CuSO_4_·5H_2_O, 10; MnSO_4_·H_2_O, 45; CoCl_2_·6H_2_O, 50; Na_2_SeO_3_, 20; H_2_CaIO_4_, 60; zeolite powder, 13485. ^5^Attractant, glycine and betaine. ^6^Tributyrin was provided by Shanghai Menon Animal Nutrition Technology Co., Ltd., Shanghai, China.

**Table 4:** Primers used in the quantitative real-time PCR analysis^1^.

Gene	Forward (5′-3′)	Reverse (5′-3′)	AE	Reference
*tnf-α*	ACACCTCTCAGCCACAGGAT	CCGTGTCCCACTCCATAGTT	0.97	[53]
*il-1β*	CATCTGGAGGCGGTGGAGGA	GGGACAGACCTGAGGGTGGT	0.98	[53]
*il-6*	CGACACACCCACTATTTACAAC	TCCCATTTTCTGAACTGCCTCT	0.95	[53]
*il-10*	AGTCGGTTACTTTCTGTGGTG	TGTATGACGCAATATGGTCTG	1.01	[53]
*ifn-γ*	TCAGACCTCCGCACCATCA	GCAACCATTGTAACGCCACTTA	0.99	[53]
*nrf2*	GATGGAAATGGAGGTGATGC	CATGTTCTTTCTGTCGGTGG	0.97	[54]
*keap1*	AACTCCAGTCTGTCTTCCCGAATC	GCGTTAATGGCACTTTGAACTCTAC	0.99	[54]
*β-Actin*	GACCTGACAGACTACCTCATG	AGTTGAAGGTGGTCTCGTGGA	1.06	[53]

^1^AE: amplification efficiency; *tnfα*: tumor necrosis factor *α*; *il-1β*: interleukin-1*β*; *il-6*: interleukin-6; *il-10*: interleukin-10; *ifnγ*: interferon *γ; nrf2*: nuclear factor erythroid 2-related factor 2; *keap1*: Kelch-like ECH-associated protein 1.

**Table 5:** Effects of tributyrin supplementation on whole body proximate composition (wet weight, %) in juvenile large yellow croaker fed diets with high level of *Clostridium autoethanogenum* protein replacing fish meal (means ± SEM, *n* = 3)^1^.

Index	Diets						
	FM	FC	TB0.05	TB0.1	TB0.2	TB0.4	TB0.8
Moisture	74.29 ± 0.17	75.67 ± 0.94	74.45 ± 0.59	75.53 ± 0.36	75.36 ± 0.98	75.04 ± 0.90	75.45 ± 0.65
Crude protein	15.62 ± 0.67	15.06 ± 0.50	15.86 ± 0.65	15.59 ± 0.43	15.36 ± 0.38	16.16 ± 0.85	16.18 ± 0.28
Crude lipid	6.53 ± 0.45	7.05 ± 0.23	6.42 ± 0.34	6.41 ± 0.27	6.47 ± 0.35	6.27 ± 0.23	6.18 ± 0.24

^1^Data sharing the same superscript letter in the common row indicated no significant difference determined by Tukey's test (*P* > 0.05).

**Table 6:** Effects of tributyrin supplementation on serum indexes of juvenile large yellow croaker fed diets with high level of *Clostridium autoethanogenum* protein replacing fish meal (means ± SEM, *n* = 3)^1^.

Index	Diets						
	FM	FC	TB0.05	TB0.1	TB0.2	TB0.4	TB0.8
ALP (U/L)	15.58 ± 1.12	14.51 ± 0.74	16.20 ± 0.48	16.80 ± 0.65	15.28 ± 0.39	16.49 ± 0.72	16.43 ± 0.41
ALT (U/L)	5.64 ± 0.27^bc^	7.97 ± 0.55^a^	4.43 ± 0.57^bc^	3.42 ± 0.74^c^	5.40 ± 0.50^bc^	6.02 ± 0.21^ab^	6.64 ± 0.48^ab^
AST (U/L)	9.24 ± 0.83^b^	15.49 ± 0.86^a^	10.32 ± 0.54^b^	9.42 ± 0.70^b^	9.74 ± 0.21^b^	9.58 ± 0.29^b^	10.36 ± 1.10^b^

^1^Data sharing the same superscript letter in the common row indicated no significant difference determined by Tukey's test (*P* > 0.05). ALP: alkaline phosphatase; ALT: alanine transaminase; AST: aspartate transaminase.

**Table 7:** Effects of tributyrin supplementation on intestinal digestive enzyme activity of juvenile large yellow croaker fed diets with high level of *Clostridium autoethanogenum* protein replacing fish meal (means ± SEM, *n* = 3)^1^.

Index	Diets						
	FM	FC	TB0.05	TB0.1	TB0.2	TB0.4	TB0.8
AMS (U/mg protein)^2^	0.54 ± 0.03	0.45 ± 0.02	0.47 ± 0.01	0.42 ± 0.01	0.50 ± 0.05	0.52 ± 0.02	0.44 ± 0.03
LPS (U/g protein)^2^	4.30 ± 0.41^ab^	2.95 ± 0.66^b^	4.42 ± 0.65^ab^	6.64 ± 0.72^a^	5.28 ± 0.37^ab^	3.84 ± 0.16^b^	3.13 ± 0.57^b^
Trypsin (U/mg protein)	4171.44 ± 99.20^b^	4135.28 ± 152.73^b^	5378.27 ± 332.62^a^	5329.12 ± 373.56^a^	4614.65 ± 156.33^ab^	4123 ± 206.87^b^	4448.29 ± 236.64^b^

^1^Data sharing the same superscript letter in the common row indicated no significant difference determined by Tukey's test (*P* > 0.05). ^2^AMS: *α*-amylase; LPS: lipase.

**Table 8:** Effects of tributyrin supplementation on activities of MDA content and antioxidant enzymes in the intestine of juvenile large yellow croaker fed diets with high level of *Clostridium autoethanogenum* protein replacing fish meal (means ± SEM, *n* = 3)^1^.

Index	Diets						
	FM	FC	TB0.05	TB0.1	TB0.2	TB0.4	TB0.8
T-AOC (U/mg protein)^2^	0.41 ± 0.02^bc^	0.39 ± 0.02^c^	0.52 ± 0.02^a^	0.49 ± 0.02^ab^	0.43 ± 0.01^bc^	0.46 ± 0.02^abc^	0.44 ± 0.02^abc^
SOD (U/mg protein)^2^	91.93 ± 3.39^ab^	87.24 ± 3.45^b^	97.8 ± 2.29^ab^	102.69 ± 1.81^a^	92.96 ± 3.45^ab^	92.04 ± 1.49^ab^	88.36 ± 2.04^b^
MDA (nmol/mg protein)^2^	1.66 ± 0.28^c^	4.53 ± 0.32^a^	2.76 ± 0.18^bc^	2.22 ± 0.23^bc^	3.06 ± 0.45^abc^	2.80 ± 0.53^bc^	3.51 ± 0.16^ab^
CAT (U/mg protein)^2^	14.39 ± 0.56^a^	13.67 ± 0.72^ab^	14.51 ± 0.31^a^	11.62 ± 0.33^b^	10.36 ± 0.64^bc^	11.63 ± 0.89^b^	8.97 ± 0.13^c^

^1^Data sharing the same superscript letter in the common row indicated no significant difference determined by Tukey's test (*P* > 0.05). ^2^T-AOC: total antioxidant capacity; SOD: superoxide dismutase; MDA: malondialdehyde; CAT: catalase.

## Data Availability

The data that supported the findings of this study are available from the corresponding author upon reasonable request.
